# A rare case of a mid sigmoid tumour presenting as an intussuscepting low rectal tumour causing clinical dilemma in a 22-year-old: a case report

**DOI:** 10.4076/1757-1626-2-8535

**Published:** 2009-08-26

**Authors:** Talal Al-Jabri, Yves Van Roon, Dhananjay Kulkarni, Tony Davies

**Affiliations:** Department of Surgery, Queen Mary’s HospitalSidcup, Kent DA146LTUK

## Abstract

A 22-year-old man presented to clinic with a 1 year history of bloody diarrhoea and weight loss. Flexible sigmoidoscopy showed the presence of a low polypoidal rectal carcinoma. Whilst awaiting neoadjuvant chemo-radiotherapy, the patient presented to accident and emergency with an anal protrusion of the tumour. An emergency laparotomy unexpectedly revealed a mid sigmoid tumour which had intussuscepted through the anus and therefore required an anterior resection as opposed to an abdomino-perineal resection. Colorectal carcinoma presents in a specifically unique pattern in patients less than 30 years. We present this rare case with a brief review of the literature.

## Introduction

The majority of rectosigmoid carcinomas are primary adenocarcinomas. Some of the most common risk factors for the development of rectosigmoid carcinomas include age, a family or personal history of colorectal carcinoma, smoking, a personal history of inflammatory bowel disease (IBD) and suffering specific genetic conditions e.g. hereditary non-polyposis colorectal carcinoma (HNPCC) or familial adenomatous polyposis (FAP). The incidence of colorectal carcinoma (CRC) is greatest in patients older than 50 years with 90% of patients presenting in the seventh decade [1]. It is very rare for rectosigmoid adenocarcinomas to develop in patients aged less than 25 years who have no predisposing factors.

Furthermore, only 5-16% of all cases of intussusception occur in adults [2]. Colonic carcinoma is considered a rare cause of intussusception. We report a case of a 22-year-old gentleman who presented with a sporadic mid sigmoid carcinoma which had intussuscepted through the anus. A brief review of the literature is included.

## Case presentation

A 22-year-old white British gentleman presented to clinic with a 1 year history of sporadic, bloody diarrhoea and a recent weight loss of 1 stone. He reported no medical antecedents, except for well-controlled asthma. In his family history; his maternal great-grandfather died of CRC aged 60. Physical examination was unremarkable. Blood tests revealed a microcytic anaemia and a raised erythrocyte sedimentation rate (ESR) with the carcinoembryonic antigen levels being normal (2 μg/L).

With the suspicion of inflammatory bowel disease, a flexible sigmoidoscopy was carried out revealing the presence of a large, polypoidal low rectal tumour almost filling the entire lumen. Biopsies confirmed the diagnosis of a moderately differentiated adenocarcinoma.

Staging computed tomography (CT) of the chest, abdomen and pelvis showed prominent, concentric thickening of the rectal mucosa. There was no evidence of lymphadenopathy or metastatic disease. In order to further identify the origin and extent of the lesion visualized on CT, an MRI was done and this showed a 10 cm tumour extending to the rectosigmoid junction without extension into the mesocolon.

**Figure 1.**
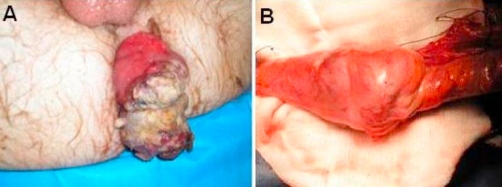
**(A)** This shows the anal protrusion presented in A&E. **(B)** The mid sigmoid tumour which intussuscepted through the anus.

The treatment plan of neoadjuvant chemo-radiotherapy followed by an abdominoperineal (AP) resection was made. Whilst awaiting treatment, the patient presented to A&E with an irreducible anal protrusion of the tumour (Figure 1A). An emergency laparotomy was performed and this unexpectedly revealed a mid sigmoid tumour intussuscepting through the anus (Figure 1B). The surgeon therefore carried out an anterior resection of the rectum, a sigmoid colectomy and a sutured anastomosis with a covering loop ileostomy. Post-operatively, the patient made a good recovery and was discharged within 5 days.

The final histology demonstrated a mid sigmoid moderately differentiated adenocarcinoma infiltrating through the muscularis propria. No extramural lymphovascular invasion was seen and the resection margins were clear of tumour. None of the 22 lymph nodes sampled showed evidence of metastases. A diagnosis of a mid sigmoid moderately differentiated adenocarcinoma, T3 N0 M0, Dukes stage B was made. Immunophenotypically, the tumour cells showed strong nuclear staining for MLH1, MSH2, MSH6 and PMS2. Therefore the possibility of HNPCC was unlikely. There was normal expression of DNA mismatch repair proteins and no known mutations in the DNA mismatch repair genes. Genetic studies excluded the possibility of HNPCC or FAP as causal factors for the tumour developing.

Neoadjuvant chemotherapy using Fluorouracil (5-FU) and Folinic Acid was well tolerated (with only a grade 1 mucositis developing). Having completed his chemotherapy regimen, the patient underwent a completion colonoscopy (normal findings) and a reversal of the defunctioning ileostomy. The screening colonoscopy of the patient’s family (father and brother) revealed no abnormalities. The patient is now on a regular colorectal carcinoma followup.

## Discussion

The vast majority of rectosigmoid carcinomas are primary adenocarcinomas. Predisposing factors include increasing age, a family history, a personal history, IBD, a high-fat diet, smoking and specific genetic conditions (e.g. HNPCC or FAP).

Approximately, 1.2-5% of CRCs present in patients aged less than 40-years [3] with 1.2% of the cases being under 30 years [4]. Rectosigmoid adenocarcinomas appear to be very rare in young adults aged less than 25 in whom there is no predisposition. However, of the cases found in the literature there are a few common clinical patterns. We briefly discuss the timing of diagnosis, symptoms at presentation, tumour staging, histology, location and prognostic features of sporadic CRCs in young patients. We then relate this to our patient.

In the majority of young patients with CRCs the diagnosis is usually made after a considerable delay from the onset of symptoms. Numerous studies show that the average delay in diagnosis since the onset of symptom is approximately 6.2 months [5-11]. Our patient presented 1 year after the onset of bloody diarrhea. This delay in diagnosis and presentation may be related to a feeling of embarrassment and awkwardness in young patients [6]. However further studies should also attempt to account for a lack of access to healthcare.

The most frequent symptoms of CRCs in young patients were rectal bleeding (46%) and abdominal pain (55%). This is also consistent with our patient who presented with a 1 year history of rectal bleeding [6].

The stage at diagnosis is specifically distinct in adults with an increasing incidence of Dukes stages A and B tumours with age. Approximately 49% of Dukes A and B tumours occur in patients over 80 years old. Conversely, Dukes C and D tumours are more common in the younger age groups [12]. Contrary to the literature our patient had a Dukes stage B tumour which is unusual for a 22 year old.

A similar pattern of an increasing incidence of poorly differentiated tumours in young patients is well documented in the literature. Once more, our patient was found to be unique in having a moderately differentiated tumour [8].

The most common location for CRCs in young patients is the rectum and sigmoid colon (including the rectosigmoid junction [6]. Approximately 54% of CRCs are estimated to occur at this location. The transverse colon is considered to be the least frequent site for CRCs with only 11% of tumours being located here [6]. Consistent with this, our patient’s tumour was in a common region at the mid sigmoid colon.

There are some important prognostic distinctions in sporadic CRC sufferers of different age groups. Young patients with sporadic CRCs usually have a poorer prognosis than elderly patients and this may be related to their delay in diagnosis. Further research is required into why this may be and it is vital that awareness regarding the distinct characteristics of sporadic CRCs in young patients is highlighted. The value of screening and improving identification of potential patients is seen in a similar context in the case of the Amsterdam and Bethesda criteria which greatly helped screen and treat individuals with familial colon carcinoma syndromes. It is our view that raising awareness of these sporadic CRCs in the young population would help identify and treat potential patients.

## Abbreviations

5-FU: fluorouracil
A&E: accident and emergency
CRC: colorectal carcinoma
CT: computed tomography
ESR: erythrocyte sedimentation rate
FAP: familial adenomatous polyposis
HNPCC: hereditary non-polyposis colorectal carcinoma
IBD: inflammatory bowel disease
MRI: magnetic resonance imaging
